# Acute unilateral vestibular loss as onset of zoster oticus infection

**DOI:** 10.1186/1471-2334-14-S7-P88

**Published:** 2014-10-15

**Authors:** Mădălina Georgescu

**Affiliations:** 1 Institute of Phono-Audiology and ENT Functional Surgery, Bucharest, Romania; 2 Carol Davila University of Medicine and Pharmacy, Bucharest, Romania

## Background

Objectives: To emphasise the importance of possible aetiologies of unilateral vestibular loss for the long term improvement of quality of life in these patients.

## Case report

A 53-year-old man presented at the emergency room with severe vertigo, vomiting and disequilibrium. Based on bed-side otoneurological evaluation and audiometry, we considered a case of vestibular neuritis (usually herpes simplex infection) and we immediately started intravenous treatment with cortisone, vestibular suppressants and betahistine.

Unfortunately, after two days, patient developed ipsilateral peripheral facial palsy, ipsilateral sensorineural severe hearing loss and hyperglycaemia. Patient was included in an antidiabetes treatment protocol. We considered at that later moment the infection as a herpes zoster infection (herpes zoster oticus or auricular herpes zoster, Ramsay Hunt syndrome) and we continued intravenous administration of vasodilators and group B vitamins, but without cortisone due to hyperglycaemia.

Facial palsy recovered slowly but completely as well as dizziness (4 month), but this was not the case with the hearing loss.

## Conclusion

Even though recommended treatment for vestibular neuritis is based on cortisone, we always have to keep in mind that the aetiology of the acute unilateral vestibular loss can make the difference in long term recovery of these patients, as well as possible risk of developing iatrogenic hyperglycaemia.

## Consent

Written informed consent was obtained from the patient for publication of this Case report and any accompanying images. A copy of the written consent is available for review by the Editor of this journal.

